# Outlines of Military Surgery

**Published:** 1840-01

**Authors:** 


Art. V.—*Outlines of Military Surgery.* By Sir George Ballingall, m.d., f.r.s., Regius Professor of Military Surgery in the University of Edinburgh. Second Edition.—*Edinburgh,* 1838. 8vo, pp. 542.

This is an improved edition of a work which ought to be in the hands of every naval and military medical officer. It is divided into three parts: "The first embraces numerous topics connected with the formation, discipline, and economy of armies. . . . The second comprises those surgical accidents and diseases peculiarly incident to military and naval men. . . . The third embraces the consideration of the most important diseases incident to troops on foreign stations." Of these departments, the second is by far the most important and complete. It forms one of the best practical manuals of all the important *incidents* of surgery anywhere to be met with; and contains the most recent and best information on all the subjects treated of. The first division is also full of interesting matter, essential to be known by the young officer. The last part is the most imperfect, and might, almost, be omitted without loss. If the author wishes to improve the details in this branch of his subject, he will find invaluable materials in Major Tulloch's Reports.

